# The low-affinity phosphate transporter PitA is dispensable for *in vitro *growth of *Mycobacterium smegmatis*

**DOI:** 10.1186/1471-2180-9-254

**Published:** 2009-12-10

**Authors:** Susanne Gebhard, Nandula Ekanayaka, Gregory M Cook

**Affiliations:** 1 Department of Microbiology and Immunology, Otago School of Medical Sciences, University of Otago, PO Box 56, Dunedin, New Zealand; 2 KIT Research Group 11-1, Institute of Applied Life Sciences, Karlsruhe Institute of Technology (KIT), Fritz-Haber-Weg 4, 76131 Karlsruhe, Germany; 3 Current address: Department Biology I, Microbiology, Ludwig-Maximilians-University Munich, Großhaderner Str 2-4, 82152 Planegg-Martinsried, Germany

## Abstract

**Background:**

Mycobacteria have been shown to contain an apparent redundancy of high-affinity phosphate uptake systems, with two to four copies of such systems encoded in all mycobacterial genomes sequenced to date. In addition, all mycobacteria also contain at least one gene encoding the low-affinity phosphate transporter, Pit. No information is available on a Pit system from a Gram-positive microorganism, and the importance of this system in a background of multiple other phosphate transporters is unclear.

**Results:**

The aim of this study was to determine the physiological role of the PitA phosphate transporter in *Mycobacterium smegmatis*. Expression of *pitA *was found to be constitutive under a variety of growth conditions. An unmarked deletion mutant in *pitA *of *M. smegmatis *was created. The deletion did not affect *in vitro *growth or phosphate uptake of *M. smegmatis*. Expression of the high-affinity transporters, PstSCAB and PhnDCE, was increased in the *pitA *deletion strain.

**Conclusion:**

PitA is the only low-affinity phosphate transport system annotated in the genome of *M. smegmatis*. The lack of phenotype of the *pitA *deletion strain shows that this system is dispensable for *in vitro *growth of this organism. However, increased expression of the remaining phosphate transporters in the mutant indicates a compensatory mechanism and implies that PitA is indeed used for the uptake of phosphate in *M. smegmatis*.

## Background

Uptake of phosphate by bacteria most commonly occurs via two systems, the low-affinity, constitutively expressed Pit system, and the high-affinity, phosphate-starvation induced Pst system [1,2]. Pit systems consist of a single membrane protein, encoded by *pitA *or *pitB*, and are energized by the proton motive force [2,3]. Pst systems are multi-subunit ABC transporters, usually encoded by a four-gene operon, *pstSCAB *[1,2]. Several bacterial species also contain additional transporters for the uptake of alternative phosphorus-compounds. Examples include the Ptx and Htx systems of *Pseudomonas stutzeri*, which transport phosphonates, phosphite and hypophosphite [4,5], and the Phn-system for the uptake of phosphonates in *E. coli *and several other Gram-negative bacteria [6-8].

Mycobacteria appear unique in that they contain several copies of high-affinity systems specific for phosphate: In the pathogenic species, such as *M. tuberculosis*, *M. bovis *and *M. leprae*, this is due to duplication of the *pst *genes [9]. For example, *M. tuberculosis *contains three different copies of *pstS*, two copies each of *pstC *and *pstA*, and one copy of *pstB *[10], plus a homologous gene, *phoT*, which has been shown to fulfill the same function as *pstB *in *M. bovis *[11]. Expression of all three copies of *pstS *under phosphate-limited conditions has been shown for *M. bovis *BCG [9], although a recent microarray analysis of phosphate-limited *M. tuberculosis *only found one of the *pst*-operons to be upregulated [12].

The environmental species *M. smegmatis *possesses only a single copy of the *pst*-operon, but it also contains a second operon, *phnDCE*, which encodes another phosphate-specific high-affinity transporter [13]. Furthermore, a third, as yet unidentified, high-affinity phosphate transport system may be present in *M. smegmatis*, because a *phnD*/*pstS *double deletion mutant still retained phosphate uptake activity with a K_m_-value of around 90 μM, which is similar to the values of the Pst and Phn systems [13].

Despite this abundance of high-affinity transporters, all mycobacterial genomes available to date also contain a *pitA *gene, encoding the low-affinity system, with the genome of *M. tuberculosis *containing a second Pit system, encoded by *pitB *[14]. The present study was directed at investigating the role of the low-affinity phosphate transporter in a bacterium containing at least two high-affinity systems, using the model of *M. smegmatis*.

## Results and Discussion

### PitA is constitutively expressed

**Figure 1:**
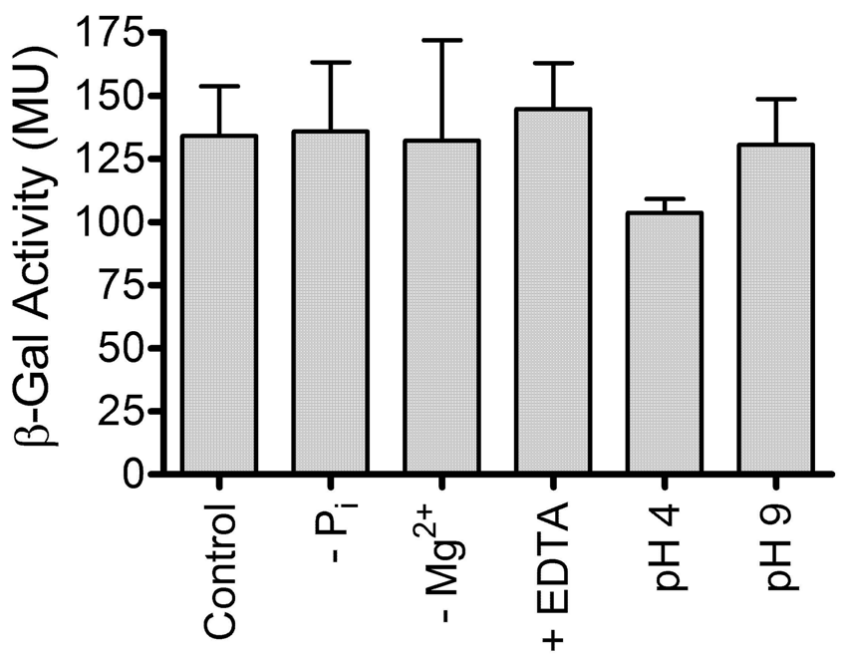
**Expression of a transcriptional *pitA-lacZ *fusion construct in *M. smegmatis***. Wild-type *M. smegmatis *harbouring the *pitA-lacZ *construct pAH1 was grown in ST medium containing 100 mM phosphate (Control), followed by 2 h starvation in phosphate-free (-P_i_) or Mg^2+^-free (-Mg^2+^) ST medium, or 2 h exposure to 5 mM EDTA (+ EDTA), pH 4 or pH 9. β-Galactosidase (β-Gal) activities were assayed and are expressed in Miller Units (MU). Results are the mean ± standard deviation of three independent experiments.

Previous studies of Pit systems have focused on Gram-negative bacteria, where *pitA *expression is independent of phosphate concentrations [1,15], while *pitB *of *E. coli *and the *pit*-like gene of *Sinorhizobium meliloti *are repressed at low phosphate concentrations [16,17]. To study the expression of *M. smegmatis pitA*, a low-copy number transcriptional *pitA*-*lacZ *fusion (pAH1) was introduced into wild-type *M. smegmatis*. The resulting strain had β-galactosidase activities of about 135 Miller Units (MU), both when grown in ST medium containing 100 mM phosphate and after 2 h starvation in phosphate-free ST medium (Figure 1). Pit systems of Gram-negatives recognize a metal-phosphate complex (MeHPO_4_) as substrate [18,19]. It was therefore possible that expression of *M. smegmatis pitA *was regulated by the availability of such MeHPO_4 _complexes, free divalent cations (e.g. Mg^2+^) or pH, as the latter influences the distribution of the different phosphate species in solution [19]. We tested the *pitA-lacZ *reporter strain after 2 h incubation in Mg^2+^-free ST medium, exposure to 5 mM EDTA, or incubation in ST medium buffered to pH 4 or pH 9. Under all conditions tested β-galactosidase activities were in the range between 100 MU and 150 MU (Figure 1). No significant differences to the control condition were observed (p > 0.05 in a one-way ANOVA test followed by Dunnett's post-test analysis), suggesting that expression of *M. smegmatis pitA *was constitutive under all conditions tested.

### A *pitA *deletion mutant has no growth defect *in vitro*

**Figure 2:**
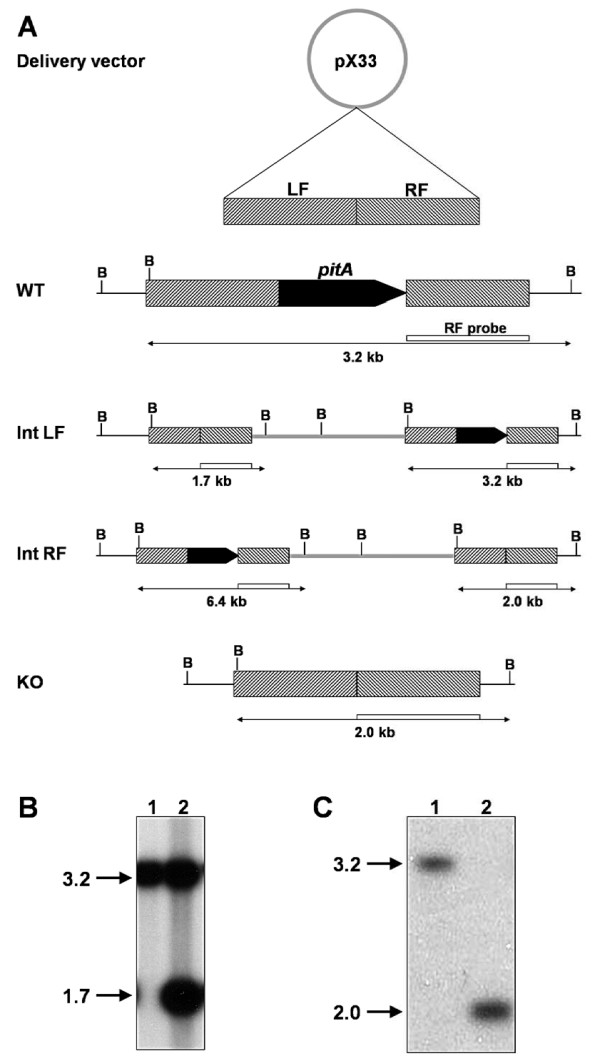
**Construction of an unmarked *pitA *deletion mutant of *M. smegmatis *mc^2^155**. A: Schematic diagram of the two-step approach for deletion of *pitA*. The knock-out construct consisted of two fragments flanking *pitA *on the left (LF) and right (RF) in pX33. Integration of the vector (thick grey line) into the chromosome (thin black line) via the left flank (Int LF) or right flank (Int RF) and subsequent deletion of *pitA *(KO) are shown. Restriction sites of BamHI (B) and fragment sizes as detected in Southern hybridization are indicated. Drawing not to scale. WT, wild-type. B: Southern hybridization analysis of the integration event. BamHI-digests of genomic DNA of wild-type mc^2^155 (lane 1) and a candidate colony (lane 2) were probed with radiolabeled right flank PCR product of the deletion construct. C: Southern hybridization analysis of *pitA *deletion. Analysis of wild-type mc^2^155 (lane 1) and the *pitA *deletion strain (lane 2) was performed as in panel B. Molecular masses are indicated in kb.

To determine if *pitA *played a role in growth and phosphate uptake of *M. smegmatis*, we next constructed an unmarked *pitA *deletion strain by an adaptation of the two-step protocol used previously to create a double-kanamycin marked mutant of *M. smegmatis *[20] (Figure 2). In the first step of mutagenesis, the construct was integrated into the chromosome by growth at 40°C. Southern hybridization analysis showed that correct integration had occurred via a cross-over event in the left flank (Figure 2B). Excision of the plasmid backbone through a second cross-over event was then selected for by growth on 10% sucrose. This second cross-over could lead either to reversion to wild-type or to deletion of the target gene. Nine colonies were screened by Southern hybridization, of which four had reverted back to the wild-type pattern, while five displayed the correct band pattern of a *pitA *deletion mutant (Figure 2C). One of the latter was chosen for further characterization.

Growth experiments showed no difference between wild-type and *pitA *mutant in LBT medium or ST medium, either under phosphate-replete conditions (100 μM to 100 mM phosphate) or phosphate-limited conditions (10 μM or 50 μM phosphate) (not shown). This characteristic of the *pitA *mutant is markedly different from the previously created *M. smegmatis *mutants in the high-affinity phosphate transporters, which were unable to grow in minimal medium at 10 mM phosphate or below [13]. As mentioned above, Pit systems of Gram-negative bacteria transport a metal-phosphate complex. While no information regarding their substrate is available for Pit systems of Gram-positives, a mutant of *Bacillus subtilis *carrying an uncharacterized mutation in phosphate uptake was also defective in uptake of metal ions [21], suggesting an interrelation between uptake of phosphate and metals. The biological role of Pit in a bacterium with a plethora of high-affinity phosphate transporters may therefore be in uptake of divalent metal ions. To test this, we performed growth experiments in Mg^2+^-limited ST medium (2 μM to 2 mM MgCl_2_), but could not discern a difference between the *pitA *and wild-type strain (not shown). Because the distribution of MeHPO_4 _versus free phosphate depends on the medium pH, with MeHPO_4 _being the predominant species at high pH values [19], it was conceivable that the physiological role of Pit is to act under conditions where most phosphate is present as MeHPO_4_. To simulate such a condition *in vitro*, we modified the ST medium to contain a high concentration of MgCl_2 _(8 mM) and low concentration of phosphate (100 μM) and adjusted the pH to 8.5 (buffered with 100 mM Tricine). No difference was found between the wild-type and *pitA *mutant strains (not shown). An *E. coli pitA *mutant displayed increased resistance to toxic divalent cations (Zn^2+ ^and Cd^2+^), due to reduced uptake of these ions [22]. The *M. smegmatis pitA *mutant and wild-type strain were therefore grown on solid media (ST agar, 50 mM MES [pH 7], 1 mM phosphate) containing 1-15 mM ZnSO_4 _or CuSO_4_. Both strains were able to grow in the presence of 1 mM of either salt, but could not grow at concentrations of 5 mM or higher. Taken together, the data presented here suggest that either PitA of *M. smegmatis *does not transport MeHPO_4_, or that one or both of the high-affinity systems also recognize such a complex as substrate. It should be noted that no substrate specificities have been determined to date for a Pst system from a Gram-positive bacterium, or for a Phn system.

### The *pitA *mutant displays no defect in phosphate uptake

**Figure 3:**
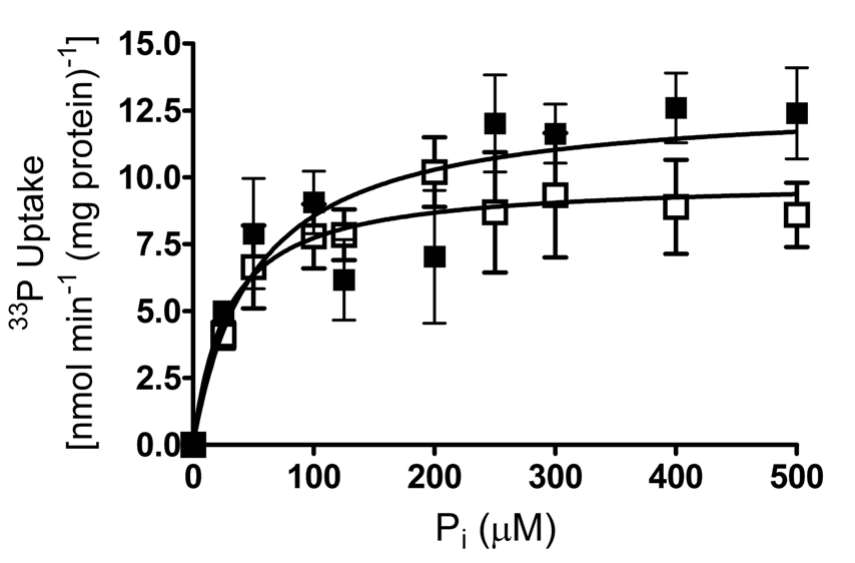
**Kinetics of phosphate uptake**. Initial uptake rates of ortho-phosphate (^33^P, > 92.5 TBq mmol^-1^) into LBT-grown whole cells of *M. smegmatis *mc^2^155 (solid squares) and the *pitA *deletion strain (open squares) were measured over 60 s at phosphate concentrations between 25 μM and 500 μM. Rates are expressed as nmol phosphate min^-1 ^mg mycobacterial protein extract^-1^, and data are shown as the mean ± standard error of the mean from two to five independent measurements per point.

We next determined the rates and kinetics of uptake of [^33^P]ortho-phosphate, to assess whether the *pitA *deletion strain had a defect in phosphate uptake. To prevent induction of the Pst or Phn systems, cells were grown in LBT medium as described in the methods section. As shown in figure 3, maximum uptake rates were 12.9 ± 1.6 nmol min^-1 ^mg protein^-1 ^for the wild-type, and 9.9 ± 1.0 nmol min^-1 ^mg protein^-1 ^for the *pitA *strain. K_d _values were similar between the strains, with 50.1 ± 26 μM phosphate for the wild-type and 27.9 ± 16.4 μM phosphate for the *pitA *strain. Slight differences in transport rates at the higher phosphate concentrations were not significant (p > 0.2 in unpaired, two-tailed t-test).

These kinetic parameters suggest that the rates of transport determined are due to activity of the high-affinity systems, because K_d _values of phosphate uptake under phosphate-starved (i.e. Pst and Phn systems induced) conditions were found to be between 40 and 90 μM phosphate [13]. The rates of transport in the present study are about ten-fold lower than those in phosphate-starved cells, consistent with the previously described 20-fold lower expression from the *pst *and *phn *promoters under these conditions [13]. PitA of *M. smegmatis *therefore appears to be either not active, or to have a very low activity, which cannot be detected over the background of the high-affinity systems using the assay employed here. Considering the abundance of phosphate transport systems in *M. smegmatis*, we hypothesized that loss of PitA is easily compensated for by increased use of the Pst and Phn systems.

### Deletion of *pitA *causes increased expression of the Pst and Phn systems

**Figure 4:**
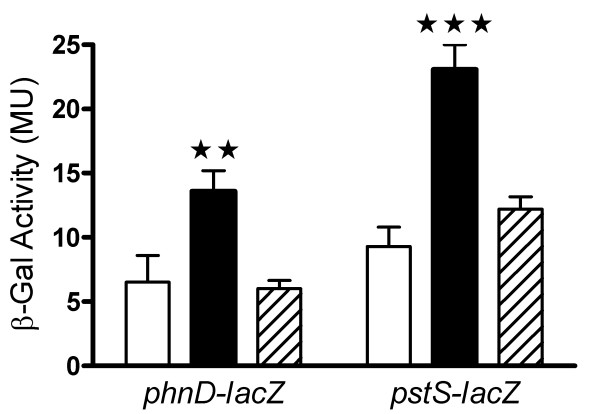
**Expression from the *pst *and *phn *promoters in the *pitA *deletion background**. Transcriptional *phnD-lacZ *and *pstS-lacZ *fusion constructs were introduced into wild-type *M. smegmatis *(open bars), the *pitA *deletion strain (black bars) and the *pitA *complemented strain (hatched bars). β-Galactosidase (β-Gal) activities, expressed as Miller Units (MU), were determined from cultures grown in ST medium with 100 mM phosphate and are shown as the mean ± standard deviation from three independent experiments. Significant differences between samples in one-way ANOVA followed by Bonferroni post-test analyses are indicated by two (p < 0.01) or three (p < 0.001) asterisks.

To address the question whether the *pitA *deletion mutant employs increased expression of either the Pst or Phn system to compensate for the deletion, we introduced the previously created transcriptional *pstS-lacZ *(pSG42) and *phnD-lacZ *(pSG10) fusion constructs [13] into the *pitA *deletion background. As shown in figure 4, under phosphate-replete conditions the activity of both promoters was increased by about two-fold in the *pitA *strain. Complementation of the deletion with a single copy of *pitA *under control of its native promoter restored expression of *pstS-lacZ *and *phnD-lacZ *to wild-type levels. No differences between strains were observed in phosphate-starved cells (data not shown). These data imply that PitA is indeed used for phosphate uptake under high phosphate conditions by *M. smegmatis*, but that loss of this system is easily compensated for by the remaining phosphate transporters.

## Conclusion

In summary, we here show that the PitA system of *M. smegmatis *is constitutively expressed under a variety of growth conditions, and that deletion of the *pitA *gene does not appear to affect growth or phosphate uptake *in vitro*. This is presumably due to compensation of the deletion by increased expression of the high-affinity phosphate transport systems, PstSCAB and PhnDCE.

The lack of phenotype of the *pitA *mutant under the growth conditions tested here, together with the wild-type levels of phosphate uptake in the mutant strain, raises the question as to why mycobacteria still contain this transporter. This point is further emphasized by the presence of a functional *pitA *gene in *M. leprae*, whose genome has undergone reductions and decay to the point where the bacterium is unable to replicate outside of its host [23]. The answer may be found in the energetics of transport: Pit systems transport metal-phosphate in symport with protons at a stoichiometry of 1:1 [3], while the Pst and Phn systems are ABC-transporters and thus likely require hydrolysis of two ATP per substrate transported [24]. Uptake of phosphate via the Pit system is therefore energetically less expensive to the cell, and this could be important under conditions where energy is limiting. However, there was no significant difference in the molar growth yield (mg [dry weight] cells/mmol of substrate consumed) between the *pitA *deletion mutant and the wild-type when grown under carbon limitation in continuous culture at a dilution rate of 0.01 h^-1 ^(doubling-time of 70 h) (our own unpublished results). We therefore hypothesize that a phenotype for a *pitA *mutant of mycobacteria may well only manifest itself *in vivo *under conditions where the cell is exposed to multiple limitations (e.g. carbon, energy, oxygen), such as are commonly found in the intraphagosomal environment of the pathogens or the soil habitat of environmental species.

## Methods

### Bacterial strains and growth conditions

All strains and plasmids used in this study are listed in Table 1. *Escherichia coli *strains were grown in Luria-Bertani (LB) medium at 37°C with agitation (200 rpm). *Mycobacterium smegmatis *strain mc^2^155 [25] and derived strains were routinely grown at 37°C, 200 rpm in LB containing 0.05% (w/v) Tween80 (LBT) or in modified Sauton's (ST) medium [13]. Variations of phosphate and MgCl_2 _concentrations and other modifications of the ST medium are given in the text. Cells to be used as inoculum in phosphate-limited ST medium were washed once in phosphate-free medium prior to use. Starvation experiments in phosphate-free ST medium were carried out as described previously [13]. *M. smegmatis *transformants were grown at 28°C for propagation of temperature-sensitive vectors and at 40°C for allelic exchange mutagenesis. Selective media contained kanamycin (50 μg ml^-1 ^for *E. coli*; 20 μg ml^-1 ^for *M. smegmatis*), gentamycin (20 μg ml^-1 ^for *E. coli*; 5 μg ml^-1 ^for *M. smegmatis*) or hygromycin (200 μg ml^-1^for *E. coli*; 50 μg ml^-1 ^for *M. smegmatis*). Solid media contained 1.5% agar. Optical density was measured at 600 nm (OD_600_) using culture samples diluted in saline to bring OD_600 _to below 0.5 when measured in cuvettes of 1 cm light path length in a Jenway 6300 spectrophotometer.

**Table 1:** Bacterial strains, plasmids and primers used in this study

Strain or Plasmid	Description^1^	Source or Reference
*E. coli*		
DH10B	F^- ^*mcrA *Δ(*mrr*-*hsdRMS*-*mcrBC*) ϕ80d *lac*Z ΔM15 Δ*lacX74 deoR recA1 araD139 *Δ(*ara leu*)7697 *galU galK rpsL endA1 nupG*	[30]
*M. smegmatis*		
mc^2^155	Electrocompetent wild-type strain of *M. smegmatis*	[25]
NP6	mc^2^155 Δ*pitA*	This study
NP13	mc^2^155 Δ*pitA *carrying pCPitA; Hyg^r^	This study
Plasmids		
pJEM15	*E. coli*-mycobacteria shuttle vector for the creation of transcriptional promoter fusions to *lacZ*; Km^r^	[27]
pX33	pPR23 [29] carrying a constitutive *xylE *marker; Gm^r^	[13]
pUHA267	*E. coli *vector with mycobacteriophage L5 integrase and *attP *for integration into L5 *attB *of mycobacteria; Hyg^r^	AgResearch, Wallaceville, NZ
pAH1	pJEM15 harbouring a 750 bp *pitA-lacZ *fusion; Km^r^	This study
pPitAKO	pX33 harbouring the *pitA *deletion construct; Gm^r^, Sac^s^, ts	This study
pCPitA	pUHA267 harbouring *pitA *with its native promoter; Hyg^r^	This study
pSG10	pJEM15 harbouring a 500 bp *phnD-lacZ *fusion; Km^r^	[13]
pSG42	pJEM15 harbouring a 560 bp *pstS-lacZ *fusion; Km^r^	[13]
Primers		
PitA1	AAATTTACTAGTGTCGTCGATGGATTCTTC	This study
PitA2	TCAGATCAGGTGAAGTCGAAAGCAAGTG	This study
PitA3	CGACTTCACCTGATCTGAAGGAACGTTGA	This study
PitA4	AAATTTACTAGTAACGAGGGTGGTAGACAGAC	This study
PitA5	AAATTTGCATGCGTGAAGTCGAAAGCAAGTG	This study
PitA6	AAATTTGTCGTCGATGGATTCTCC	This study
cPitAf	AAATTTAAGCTTGTCGTCGATGGATTCTCC	This study
cPitAr	AAATTTAAGCTTACGTTCCTTCAGATCAGAC	This study

^1^Km^r^, kanamycin resistance; Gm^r^, gentamycin resistance; Hyg^r^, hygromycin resistance; Sac^s^, sucrose sensitivity; ts, temperature sensitivity. Primer sequences are given in the 5'-3' direction; restriction sites included in the primer sequences are underlined.

### DNA manipulation and cloning of constructs

All molecular biology techniques were carried out according to standard procedures [26]. Restriction or DNA modifying enzymes and other molecular biology reagents were obtained from Roche Diagnostics or New England Biolabs. Genomic DNA of *M. smegmatis *was isolated as described previously [13]. All primer sequences are listed in Table 1.

To create a transcriptional fusion of the *pitA *promoter to *lacZ*, a fragment containing 750 bp of upstream sequence to *pitA *(MSMEG_1064) was amplified with primers PitA6 and PitA5 and cloned into the BamHI and SphI sites of the low copy-number vector (3-10 copies per cell) pJEM15 [27], resulting in plasmid pAH1. Assays for β-galactosidase activity were carried out as described previously [13]. Cells of *M. smegmatis *harbouring the empty vector pJEM15 displayed β-galactosidase activities of less than 2 MU. Statistical analysis of reporter-strain experiments after starvation or stress-exposure was performed using one-way ANOVA followed by a Dunnett's post-test comparison of each sample to the control condition. Data from experiments of the *phnD-lacZ *and *pstS-lacZ *constructs in various genetic backgrounds were analyzed by one-way ANOVA followed by Bonferroni post-test comparison of all pairs of data-sets. All statistical analyses were performed using GraphPad Prism 4 software.

To create a construct for markerless deletion of *pitA*, an 833 bp fragment flanking *pitA *on the left, including 62 bp coding sequence, was amplified with primers PitA1 and PitA2, and a 1022 bp fragment flanking *pitA *on the right, including 4 bp coding sequence, was amplified with primers PitA3 and PitA4. The two products were fused by PCR-overlap extension [28], cloned into the SpeI site of the pPR23-derived [29] vector pX33 [13], creating pPitAKO, and transformed into *M. smegmatis *mc^2^155. Deletion of *pitA *was carried out using the two-step method for integration and excision of the plasmid as described previously [20]. Correct integration and excision were confirmed by Southern hybridization analysis as described previously [13]. The deletion resulted in loss of 95% of the *pitA *coding sequence, creating strain NP6

For complementation of the *pitA *deletion, *pitA *plus 790 bp upstream DNA was PCR amplified with primers cPitAf and cPitAr and cloned as a HindIII fragment into the integrative *E. coli*/mycobacteria shuttle vector pUHA267 (AgResearch, Wallaceville), creating plasmid pCPitA. Transformation into the *pitA *mutant resulted in strain NP13.

### Phosphate transport assays

Strains of *M. smegmatis *were grown to an OD_600 _of 1 in LBT medium, collected by centrifugation and resuspended to an OD_600 _between 1.5 and 2 in pre-warmed assay buffer (50 mM MOPS [pH 7.5], 5 mM MgCl_2_, 0.05% (w/v) Tween80, 0.4% glycerol, 37°C). Initial rates of uptake of [^33^P]ortho-phosphate (> 92.5 TBq mmol^-1^; Amersham) were determined over a range of phosphate concentrations between 25 μM and 500 μM as described previously [13].

## Authors' contributions

SG contributed to design of the study, participated in growth experiments, phosphate transport and reporter gene assays and drafted the manuscript. NE carried out the molecular work and participated in all other experimental aspects. GMC contributed to design of the study, participated in phosphate transport assays and helped to draft the manuscript. All authors read and approved the final manuscript.
